# Monolithic All-Solid-State High-Voltage Li-Metal Thin-Film Rechargeable Battery

**DOI:** 10.1021/acsaem.2c01581

**Published:** 2022-09-27

**Authors:** Iñaki Madinabeitia, Jokin Rikarte, Ane Etxebarria, Giorgio Baraldi, Francisco José Fernández-Carretero, Iñigo Garbayo, Rosalía Cid, Alberto García-Luis, Miguel Ángel Muñoz-Márquez

**Affiliations:** † TECNALIA, Basque Research and Technology Alliance (BRTA), Parque Científico y Tecnológico de Gipuzkoa, Mikeletegi Pasealekua 2, 20009 Donostia-San Sebastián, Spain; ‡ Centre for Cooperative Research on Alternative Energies (CIC energiGUNE), Basque Research and Technology Alliance (BRTA), Alava Technology Park, Albert Einstein 48, 01510 Vitoria-Gasteiz, Spain; § Departamento de Física de la Materia Condensada, Facultad de Ciencia y Tecnología, Universidad del País Vasco, UPV/EHU, P.O. Box 644, 48080 Bilbao, Spain

**Keywords:** magnetron sputtering, thermal evaporation, thin-film battery, all-solid-state, LiNi_0.5_Mn_1.5_O_4_, LiPON, stainless steel current collector

## Abstract

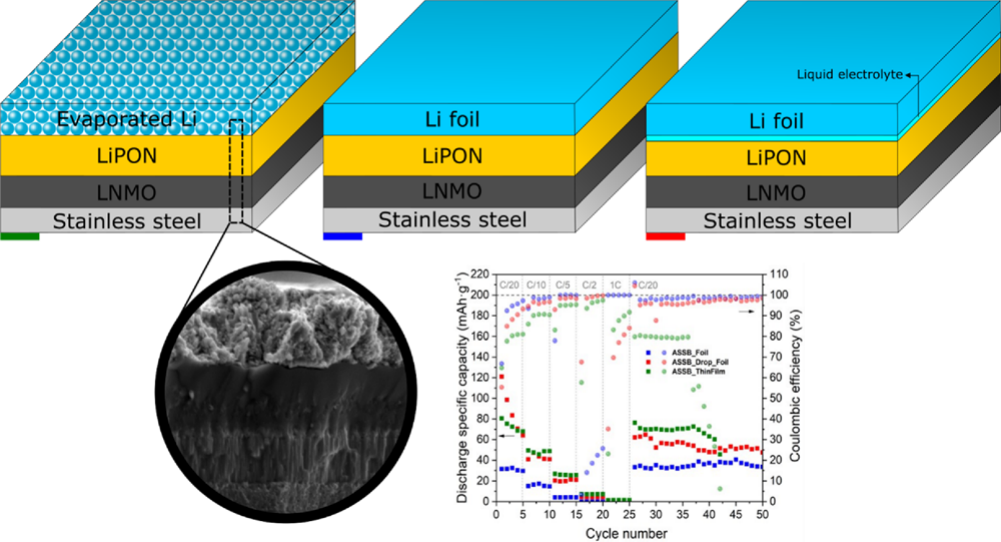

The substitution of an organic liquid electrolyte with lithium-conducting solid materials is a promising approach to overcome the limitations associated with conventional lithium-ion batteries. These constraints include a reduced electrochemical stability window, high toxicity, flammability, and the formation of lithium dendrites. In this way, all-solid-state batteries present themselves as ideal candidates for improving energy density, environmental friendliness, and safety. In particular, all-solid-state configurations allow the introduction of compact, lightweight, high-energy-density batteries, suitable for low-power applications, known as thin-film batteries. Moreover, solid electrolytes typically offer wide electrochemical stability windows, enabling the integration of high-voltage cathodes and permitting the fabrication of higher-energy-density batteries. A high-voltage, all-solid-state lithium-ion thin-film battery composed of LiNi_0.5_Mn_1.5_O_4_ cathode, a LiPON solid electrolyte, and a lithium metal anode has been deposited layer by layer on low-cost stainless-steel current collector substrates. The structural and electrochemical properties of each electroactive component of the battery had been analyzed separately prior to the full cell implementation. In addition to a study of the internal solid–solid interface, comparing them was done with two similar cells assembled using conventional lithium foil, one with thin-film solid electrolyte and another one with thin-film solid electrolyte plus a droplet of LP30 liquid electrolyte. The thin-film all-solid state cell developed in this work delivered 80.5 mAh g^–1^ in the first cycle at C/20 and after a C-rate test of 25 cycles at C/10, C/5, C/2, and 1C and stabilized its capacity at around 70 mAh g^–1^ for another 12 cycles prior to the start of its degradation. This cell reached gravimetric and volumetric energy densities of 333 Wh kg^–1^ and 1,212 Wh l^–1^, respectively. Overall, this cell showed a better performance than its counterparts assembled with Li foil, highlighting the importance of the battery interface control.

## 1 Introduction

The imminent revolution of Internet of Things (IoT), with millions of interconnected energy-demanding smart microdevices and sensors, demands the development of a new generation of high-energy-density, long-lasting powering devices, which outperform current Li-ion batteries.^1,2^ In this sense, the use of Li metal as an anode is living an intense upturn lately to profit from its high theoretical specific capacity (3,860 mAh g^–1^), the lowest negative electrochemical potential (−3.040 V vs SHE), and low density (0.59 g cm^–3^).^3,4^ However, its use still entails several challenges when combined with state-of-the-art liquid electrolytes, especially related to their (i) limited electrochemical stability window, (ii) flammability, and (iii) Li dendrite formation. In this scenario, all-solid-state Li-metal batteries (ASSBs), where the liquid electrolyte is replaced by a solid Li-ion conducting material, are attracting increasing attention in order to overcome the mentioned technological limits associated with liquid electrolytes. At the same time, widening the voltage stability window also opens up the possibility of working with high-voltage cathodes, paving the way for the fabrication of batteries with higher energy density, longer cycle life, and increased safety, as otherwise expected when using solid electrolytes.^3,5^

Two main types of materials result in mechanically and electrochemically stable Li-ion conducting solid electrolytes, namely, polymers and ceramics. On the one hand, polymer-based electrolytes have been widely reported (in particular polyethylene oxide (PEO)-based formulations) due to easy processability, low toxicity, low cost, and good chemical stability. However, they are limited by a low Li-ion conductivity at room temperature, thus forcing the cell operation at high temperatures, usually *ca.* 70 °C. On the other hand, ceramic materials are lately receiving increasing attention due to their competitive ionic conductivity, even at room temperature, as well as thermal and electrochemical stability.^5^ Moreover, the fact that in some cases solid-state batteries have operated for more than 10,000 cycles is an irrefutable proof of the inherently slower reactivity of solid electrolytes compared to liquid ones, resulting in longer device lifetime for solid-state cells.^6^ Furthermore, a particularly relevant advantage of ceramic electrolytes to this work is the possibility of downscaling them to thin-film format (<1 μm) for the development of all-solid-state microbatteries.^7^ Allowing the fabrication of batteries with capacities ranging from 0.1 to 5 mAh, ideal, e.g., for IoT applications.^2,8^

**Figure 1:**
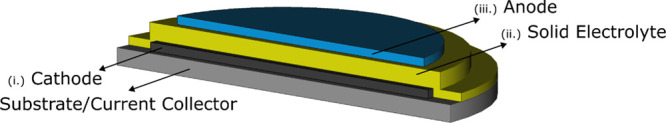
Sketch of a thin-film battery. The battery components are sequentially grown on top of a conductive substrate: (i) cathode, (ii) electrolyte, and (iii) anode. A series of shadow masks are generally employed to define the deposition areas and avoid short-circuiting both electrodes.

As shown in Figure 1, all-solid-state thin-film microbatteries can be assembled as a sequential stack of thin-film layers deposited on a substrate by means of microfabrication technologies that rely on shadow masks and selective etching processes to define the functional area of each cell component. Back in 1994, Bates and co-workers developed and patented the first thin-film microbattery, fabricated by magnetron sputtering at Oak Ridge National Laboratory (ORNL).^9^ Notably, this coating technique is still nowadays one of the most widely used techniques for processing thin-film components owing to its high deposition rates, stoichiometry control, reproducibility, solvent-free processing, and, importantly, ability to scale-up to industrial production (e.g., roll-to-roll systems).^10−12^ Its suitability and reliability to work with almost any type of material (conductor/insulator and crystalline/amorphous) has been demonstrated in the fabrication of different battery components: positive electrodes such as LiMn_2_O_4_,^13^ LiCoO_2_,^14^ and LiFePO_4_;^15^ amorphous Li_2_PO_2_N^16^ and garnet-structure like Li_7_La_3_Zr_2_O_12_^17^ solid electrolytes; negative electrodes such as Li_4_Ti_5_O_12_^18^ and SnO_2_;^19^ and metallic current collectors such as Ag, Cu, or Al.^20^

Nowadays, the portfolio of thin-film materials is very broad. Most of the studies are focused on the advanced cathode characterization, with metallic lithium as the anode and lithium phosphorous oxynitride (Li_3_PO_4–*x*_N_*x*_, commonly referred as LiPON) as the solid electrolyte. Despite the progress made in the development of new materials, nowadays the most used inorganic solid-state electrolyte material in thin-film form is LiPON. Using LiPON as an electrolyte allows widening the voltage stability window of the cells up to 5.5 V,^21^ enabling the integration of high-voltage positive electrodes^22^ and paving the way toward fabrication of high-energy-density batteries.^3^ Its stability against metallic Li and negligible electronic conductivity (>10^14^ Ω cm) are key aspects here, while its main drawback is the relatively low ionic conductivity (2×10^–6^ S cm^–1^ at room temperature).^23^ Many research efforts around this solid electrolyte have been primarily focused on increasing its ionic conductivity by varying deposition conditions, such as the sputtering power, N_2_ pressure, and substrate temperature.^24−26^ However, it is to be noted that a drastic thickness reduction of the electrolyte layer eventually permits its practical use, regardless of the lower conductivity. Finally, regarding the Li metal anode, the most widely used way to deposit the lithium thin layer is by thermal evaporation, although its deposition details or characteristics are rarely given. The most commonly used positive electrodes such as olivine LiFePO_4_, spinel LiMn_2_O_4_, or layered LiCoO_2_ have a limited working voltage of 3.5, 4.2, and 4.2 V vs Li/Li^+^, respectively.^14,15,27^ In this sense, the cobalt-free LiNi_0.5_Mn_1.5_O_4_ (LNMO) was proposed as a very promising alternative for the next generation of high-power thin-film, providing a high working voltage of 4.7 V vs Li/Li^+^ (corresponding to two redox couples of nickel) that results in a high energy density (specific capacity of *C*_theo._ = 146.6 mAh g^–1^), high charge/discharge rate capability, an acceptable electronic and Li-ion conductivities.^28−30^

In 2012, the combination of LNMO and LiPON was explored by Baggetto and co-workers at ORNL. The coating of LNMO with LiPON was found to result in the reduction of Coulombic losses and in improved rate capability when tested using LiPF_6_ dissolved in an organic carbonate-based liquid electrolyte and Li metal as the negative electrode.^31,32^ The same authors also studied the influence of metal oxide coatings on LNMO thin-film electrodes when cycled using a liquid electrolyte.^33^ Other authors have reported the electrochemical response of epitaxial LNMO (100) grown on LaNiO_3_ (100) current collectors that have been deposited on Nb-doped SrTiO_3_ (100) substrates; in this case, amorphous Li_3_PO_4_ was used as an electrolyte and Li metal as a negative electrode.^34^ So far, the best-performing LNMO thin-film cell with a LiPON electrolyte and a Li metal anode was grown on Pt-coated Al_2_O_3_ substrates.^6^ Besides LNMO, LiCoPO_4_ has been used as a positive electrode (4.8 V vs Li/Li^+^) in thin-film batteries with LiPON as the electrolyte and Li metal as the anode;^35^ in this case, though, the highest conductive phase appeared only at 2.5 V.

Despite the intensive efforts devoted toward the development of solid-state batteries and the identified gaps in terms of materials science, processing science, and design engineering,^36^ the poor interconnection of the solid–solid electrode–electrolyte interface is often cited as the main limitation for full exploitation of the electrochemical properties of electroactive materials.^3^ The poor adherence generated by discontinuities at the interface results in limited diffusion paths of Li ions^37^ that is often addressed by integrating liquid or gel catholytes in the system, thus deviating from the pure solid-state concept. Moreover, the appearance of detrimental reaction products at the inner battery interfaces, predominantly between the Li metal and the solid electrolyte, is usually behind the high internal resistance of the cell that ultimately reduces the cell performance.^38^ In this regard, it is worth mentioning that low interface resistance in solid-state thin-film batteries with the LNMO electrode and Li_3_PO_4_ electrolyte has already been demonstrated. However, the Li metal–solid electrolyte interface is still an open question.^39^

In this work, a thin-film ASSB comprising a high-voltage cathode LNMO, a LiPON ceramic electrolyte (both deposited by magnetron sputtering), and a metallic Li anode (thermally evaporated) has been developed. All was sequentially deposited on functional and cost-effective stainless-steel current collector (∼$1.5 kg^–1^), starting from the cathode. The structural and electrochemical properties of each electroactive component of the battery have been analyzed separately before the building up of the cell. The optimization of the cathode has been described in a previous work.^40^

## 2 Experimental Section

### 2.1 Cathode Fabrication

LNMO deposition was performed by a CemeCon MF-AC dual technology-based magnetron sputtering system on low-cost stainless-steel discs (316L, Hohsen Corp.) using a 12 mm circular mask. The selected growth parameters are detailed in a previous work focused on the optimization of LNMO thin-films.^40^ Under these conditions, an 8.91 nm min^–1^ deposition rate and a good film adhesion were achieved. The growth process lasted for 2 h, leading to the deposition of 1.07 ± 0.02 μm-thick films. After deposition, the LNMO film was annealed up to 600 °C for 1 h in air to achieve a desired crystallographic structure using a heating ramp of 5 °C min^–1^ and cooling down slowly inside the switched off oven.

### 2.2 Solid Electrolyte Fabrication

LiPON thin-film electrolytes were deposited by radio frequency (RF) reactive magnetron sputtering with an integrated full-face erosion technology, wherein the whole target surface is sputtered via motor-driven dynamic plasma scanning. In this way, it was possible to increase the deposition rate and homogeneity of the thin film across the deposition area. A power density of 1.09 W cm^–2^ was applied to a 3 in. Li_3_PO_4_ circular target (Toshima Manufacturing Co.). The substrate-to-target distance was 90 mm with an incident angle of 45°. During the sputtering process, a working pressure of 4.5×10^–3^ mbar of Ar and N_2_ was kept constant. In order to analyze the role of the nitrogen concentration on the solid electrolyte, different percentages of pure nitrogen (N_2_) flow rates were used: 0, 50, and 100%. These samples are henceforth referred as LiPON_0, LiPON_50, and LiPON_100, respectively. Sixteen-hour-long processes were carried out, leading to 1.3 μm-thick solid electrolyte layers.

The LiPON thin films were grown on atomically flat Si (100) single-crystal substrates for morphological, structural, and elemental characterization. SEM (FEI quanta-200FEG) was used to measure the thickness and surface morphology. The crystallographic structure study has been carried out by X-ray diffraction (XRD) using a Bruker Advance D8 diffractometer with Cu radiation (Cu Kα_1,2_ λ = 1.5406 and 1.5444 Å). The study was conducted at 2θ angles ranging from 15 to 65° 2θ with a step size of 0.02°. The resulting diffractograms were interpreted using both the diffraction data found in the literature and the Diffract.eva software. The elemental composition of the surface of phosphorous oxide and oxynitride samples was analyzed by X-ray photoelectron spectroscopy (XPS) using a Phoibos 150 spectrometer (Specs GmbH) in fixed analyzer transmission mode and a non-monochromatic Mg Kα X-ray source (*h*ν =1253.6 eV) operated at 100 W. Survey spectra were obtained with 0.5 eV step size and a pass energy of 50 eV. Particular regions of O 1s, N 1s, C 1s, P 2p, and Li 1s were acquired with high resolution: a step size of 0.1 eV and a pass energy of 30 eV. Experimental data was treated with CasaXPS software. The zero of binding energy for all the spectra was calibrated using the aliphatic carbon signal, which was set at 284.6 eV.

In order to evaluate the ionic conductivity of the electrolyte, LiPON thin films were deposited on 10 × 10 × 0.5 mm dielectric MgO (100) single-crystal substrates (CrysTec GmbH). Here, 6 × 1 mm- and 440 nm-thick copper electrodes were deposited on top of the LiPON films by means of direct current (DC) magnetron sputtering and using a shadow mask. An air-tight Linkam HFS600E-PB4 stage was used for the in-plane ionic conductivity measurements. Impedance measurements were carried out with the help of a Biologic EC-Lab potentiostat in the frequency range from 7 MHz to 100 mHz, with 10 points per decade and a sinus amplitude of 20 mV. The conductivity measurements were performed at temperatures of 150, 200, and 250 °C under a controlled Ar atmosphere within the Linkam stage.

### 2.3 Anode Fabrication

The lithium metal anode was deposited by thermal evaporation using commercial crucibles from AlfaSources (AlfaVakuo e.U.) that contained the evaporation material. Prior to the lithium deposition, the evaporation chamber was evacuated down to a base pressure below 1 × 10^–8^ mbar, the substrate-to-crucible distance was set at 50 mm, and a current of 12 A was applied to the crucible that contains the lithium. During the deposition, a working pressure of 5 × 10^–8^ mbar was maintained for a 24 h-long process that resulted in a 1 μm-thick lithium film.

For the transport and handling of the air-sensitive samples, air-tight transfer arms and an inert atmosphere glove box (Ar filled, with H_2_O and O_2_ concentrations below 0.1 ppm) were used.

### 2.4 All-Solid-State Thin-Film Battery Fabrication

The assembly of the monolithic all-solid-state battery was performed following a sequential process in which the different components were grown layer by layer. First, the LNMO thin-film cathode was deposited on top of the SS substrate using the 12 mm diameter shadow mask followed by a post-annealing process in order to achieve the desired crystallographic structure. On top of that, the LiPON thin-film electrolyte was deposited; in this case, no shadow masks were used, thus a full coverage of the LNMO/SS sample was attained. Finally, the lithium anode was deposited by thermal evaporation over the electrolyte, this time using again a circular shadow mask with a 12 mm diameter hole.

By means of an air-tight transfer tool, the resulting all-solid-state cell was transferred into an Ar-filled glove box where it was sealed in a pouch cell (image of the cell included in the last section of the Supporting Information file, Figure S7a). The finalized cells were connected to a MACCOR battery cycler, which is placed in a laboratory with controlled temperature (25 °C), to perform galvanostatic cycling. For these tests, the voltage window was in the 3.5–5 V range and the current rates were set in the C/20 to the C range. Specific capacities and currents were determined with respect to the exact mass of the positive electrode loading. The electrochemical performance data of the ASSB cell was compared with data obtained after performing the same electrochemical tests on cells assembled using conventional lithium foil as an anode and after incorporating a drop of liquid electrolyte (1 M LiPF_6_ in dimethyl carbonate (DMC) and ethylene carbonate (EC) (1:1 v/v, Solvionic, 99.9%)) in the interface between the solid electrolyte and the lithium foil.

## 3 Results and Discussion

### 3.1 Solid Electrolyte Characterization

**Figure 2:**
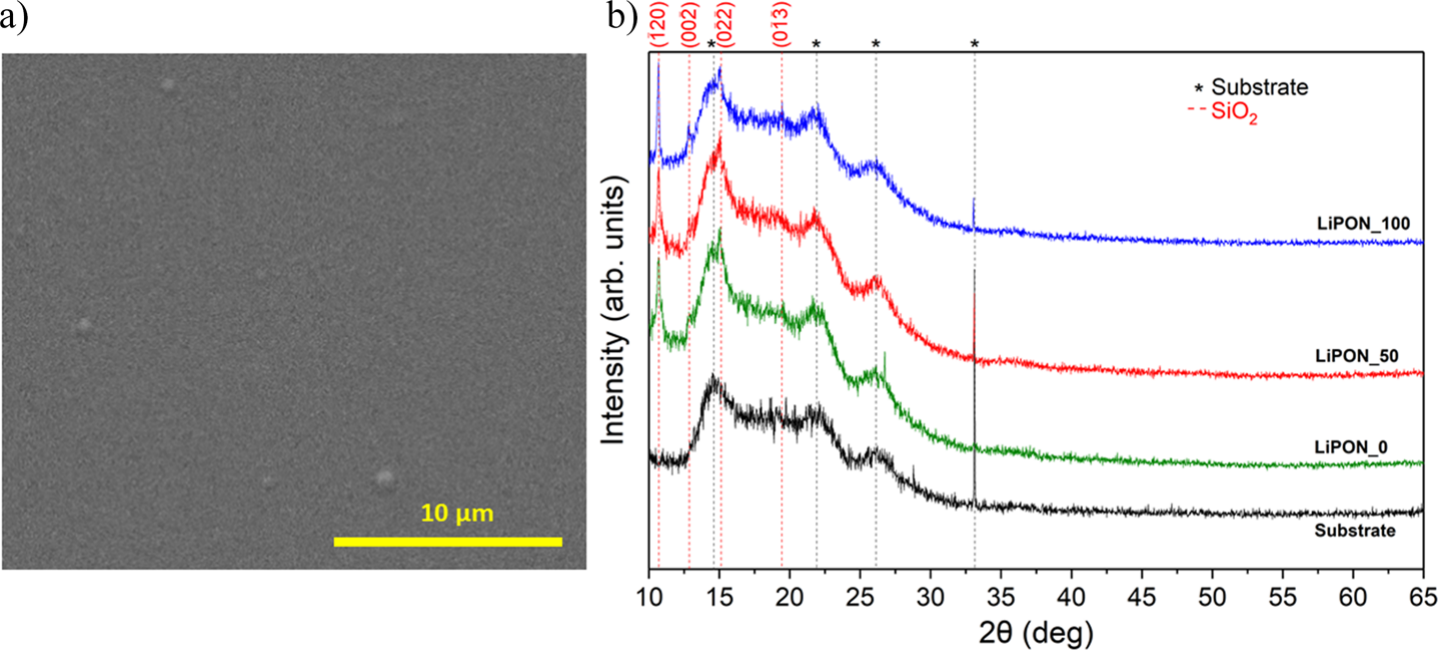
(a) Top-view SEM images of LiPON_50 thin film and (b) X-ray diffraction patterns for LiPON_0, LiPON_50, and LiPON_100.

The surface morphology of the electrolyte films has been evaluated by means of SEM. While the surface of the sample deposited only with argon is granulated, a smoother surface was obtained when increasing the nitrogen flow (Figure 2a) (images included in the Supporting Information, Figure S1). Nevertheless, from a morphological point of view, all films presented a dense and homogeneous structure required.

X-ray diffraction measurements confirmed the expected amorphous nature of the LiPON thin films prepared in this work (Figure 2b). As it can be seen in the diffraction pattern, only those peaks related to the silicon substrate and Kapton film used for sample protection (see diffraction pattern from the reference substrate in black, Figure 2b) were observed for all the samples. Meanwhile, no peaks associated with electrolyte crystallinity could be identified, confirming the amorphousness of the LiPON films. This statement is in line with the reported literature on LiPON thin-film layers deposited by RF magnetron sputtering.^41,42^ The presence of orthorhombic SiO_2_ (ref. COD 4110502) is worth mentioning,^43^ with diffraction peaks located at 10.6, 12.8, 15.1, and 18.9° 2θ, which has been formed during deposition. In any case, the SiO_2_ formation would be associated with the use of Si as the substrate and would not be present when moving to the functional SS substrate.

**Figure 3:**
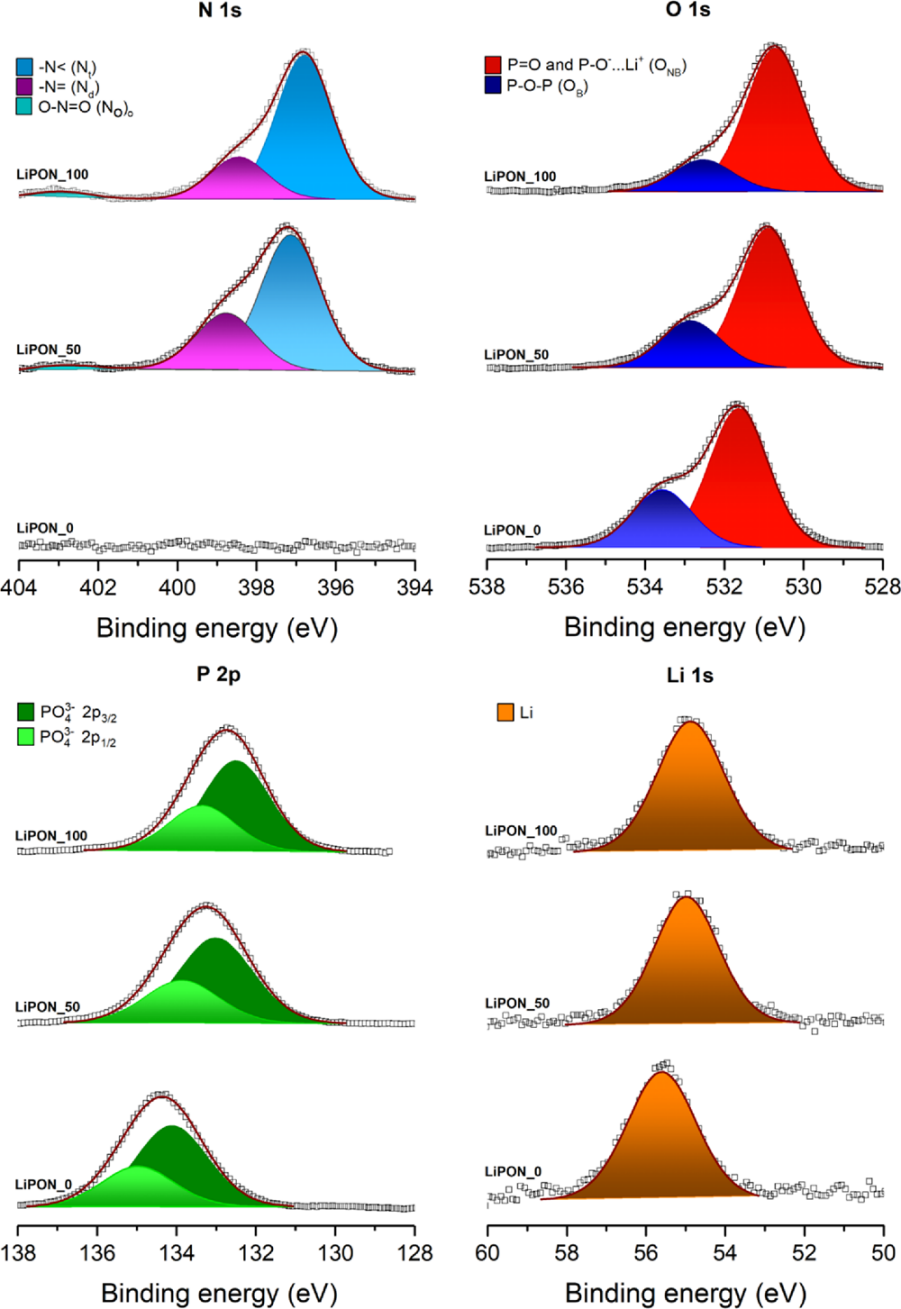
XPS profiles deconvolution of the regions O 1s, N 1s, P 2p, and Li 1s for LiPON_0, LiPON_50, and LiPON_100 samples. Note that the ordinate axes, which correspond to the normalized photoelectron intensity in arbitrary units, have not been incorporated in the graph for a better visualization.

In order to identify any change related to the insertion of nitrogen into the structure, surface elemental characterization was performed by means of XPS. Figure 3 shows the XPS spectra of the O 1s, N 1s, P 2p, and Li 1s regions for LiPON_0, LiPON_50, and LiPON_100 films.

As expected, the major difference between N-containing and N-free electrolyte films becomes visible when analyzing the N 1s region. While the film deposited using only Ar as background gas (LiPON_0) shows no N 1s peak, the other two samples are characterized by a broad N 1s photoelectron peak that can be resolved with two different components. This is in agreement with previous reports,^26,44^ where the two N 1s components are ascribed to a doubly coordinated nitrogen −N= (labeled as N_d_) and a triply coordinated nitrogen −N< (labeled as N_t_), as otherwise expected for LiPON (see Figure S2 in the SI). Additionally, a weak peak at a higher binding energy (around 403 eV) can be seen, probably corresponding to O–N=O (N_o_).^45^ In both cases, N_d_ presents a more intense signal than the N_t_ contribution, indicating a relatively higher concentration in the glass structure, with N_d_/N_t_ being about 2.39 for LiPON_50 and 3.42 for LiPON_100. Notably, an increase in doubly coordinated nitrogen is observed when the amount of nitrogen in the system is increased. When comparing the two nitrated samples, LiPON_50 and LiPON_100, a clear shift toward lower binding energies can be observed when increasing N content. This way, the doubly coordinated nitrogen shifts from 397.1 eV in the case of LiPON_50 and to 396.8 eV in the case of LiPON_100, while the less intense triply bonded nitrogen contribution shifts from 398.8 to 398.5 eV. N_o_ also presents a similar shift, from 403.0 eV for LiPON_50 to 402.90 eV for LiPON_100. This slight binding energy shift can be associated with a decrease in the surface charge load associated to the electro-insulating nature of these oxides.^46^

The analysis of the O 1s spectrum has been carried out using the model proposed by Brow et al.,^47^ where the main contribution at low binding energies (*ca*. 531 eV) corresponds to the non-bridging oxygen (O_NB_), whereas the shoulder located at high binding energies (around 533 eV) corresponds to a bridging oxygen (O_B_). As described in the literature,^48,49^ the O_NB_ contribution could be interpreted as the sum of two types of non-bridging oxygens present in lithium phosphate oxide and oxynitride structures, P=O and P–O^–^···Li^+^.

Like the abovementioned case, introducing nitrogen into the system entails a slight displacement toward low binding energies for both the non-bridging (531.6 > 530.9 > 530.7 eV for LiPON_0, LiPON_50, and LiPON_100, respectively) and bridging (533.6 > 532.8 > 532.6 eV) oxygens. Similarly, as the nitrogen is inserted in the phosphate base glass, the area of O_B_ decreases about that of O_NB_. In the sample LiPON_0, the O_B_/O_NB_ ratio is 0.42, close to the theoretical ratio of 0.50 for a metaphosphate glass;^47^ while for 50% of nitrogen, the ratio is reduced to 0.33; finally, for 100% nitrogen, it reduces to 0.22. In order to provide a theoretical estimation of the efficiency of nitrogen incorporation in the LiPON structure, two structural models will be used. The first one is related to the specific region of O 1s as proposed by Brow et al.^47,48^ (eq 1)
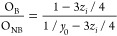
1where *y*_0_ is the O_B_/O_NB_ ratio for the non-nitridated sample (LiPON_0) and *z*_i_ is the N/P ratio of the nitridated sample under consideration. In this model, it is assumed that nitrogen has no preference in replacing either bridging or non-bridging oxygens, *i.e.*, equal numbers of these species are replaced during nitridation.

The second model, proposed by Marchand et al.,^49^ correlates the amount of doubly and triply coordinated nitrogen in the sample under consideration with the amounts of non-bridging and bridging oxygen, based on the substitution of 3 O^2–^ by 2 N^3–^ (eq 2)
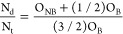
2

Table 1 presents the obtained experimental ratios of O_B_/O_NB_ as well as N_d_/N_t_ and compares them with the expected theoretical values for the analyzed samples found in the literature. As it can be observed, the values obtained from the theoretical models for a nitrated LiPON_50 and LiPON_100 films match well with the experimental ones, suggesting efficient nitrogen incorporation into the films for both cases.

**Table 1:** Experimental and Theoretical Ratios of Lithium Phosphate Oxide and Oxynitride Glasses

	O_B_/O_NB_	N_d_/N_t_	(O_B_/O_NB_)^47^ theoretical	(N_d_/N_t_)^49^ theoretical
LiPON_0	0.42			
LiPON_50	0.33	2.39	0.29	2.32
LiPON_100	0.22	3.42	0.18	3.33

A quasi-symmetrical peak was obtained in the P 2p photoelectron line for the three LiPON films, with no shape evolution upon nitridation (Figure 3). These peaks could be fitted with a main doublet, with spin-orbit splitting around 0.86 eV between 2p_3/2_ and 2p_1/2_ orbital lines. This doublet corresponds to the PO_4_^3–^ tetrahedral environment present in the lithium phosphate. However, the binding energy shift observed for the different LiPON films is more pronounced than those found in the other elements (2p_3/2_ peaks located at 134.1 > 133.0 > 132.5 eV for LiPON_0, LiPON_50, and LiPON_100, respectively) and it is believed to be nested on the replacement of P–O bonds by P–N bonds since reducing the average ionic charge on the phosphorus ions results in a decrease in the binding energy.^26,47^

**Figure 4:**
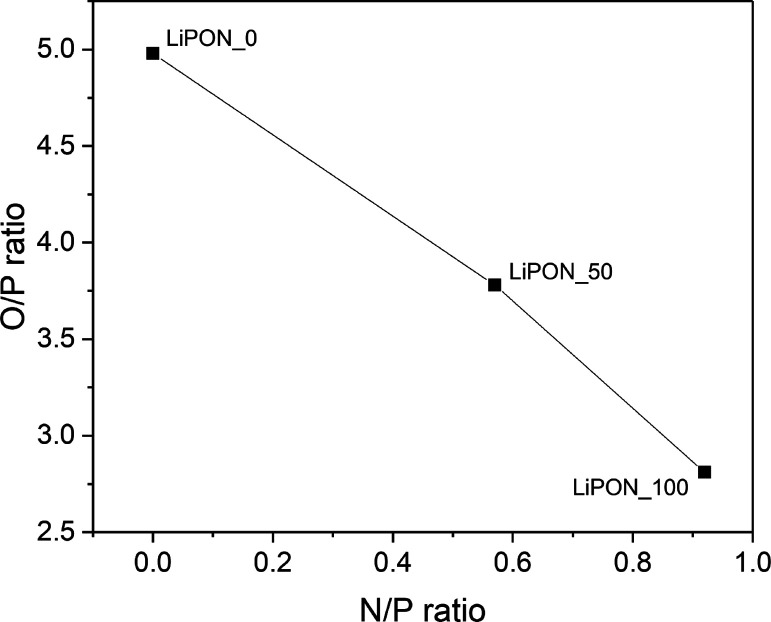
Quantitative evolution of O/P and N/P ratios with nitrogen incorporation.

Figure 4 shows the quantitative evolution of the O/P and N/P ratios with nitrogen incorporation. As it can be observed, the oxygen content decreases with the introduction of nitrogen (from 4.98 to 2.81 ratio) and vice versa in the case of nitrogen content (from 0 to 0.92). This matches well with the expected behavior: the O/P and N/P values obtained by Fleutot et al.^26^ range from 4.3 to 3.0 and from 0 to 1, respectively. Thus, this confirms the successful substitution of oxygen by nitrogen in the system, as discussed before.

Finally, looking back to Figure 3, similarly to the phosphorous case, the Li 1s spectra show a quasi-symmetrical shape. They are analyzed with a single lithium element, also undergoing a similar displacement observed in the previous cases, 55.6 > 55.0 > 54.9 eV for LiPON_0, LiPON_50, and LiPON_100, respectively.

**Figure 5:**
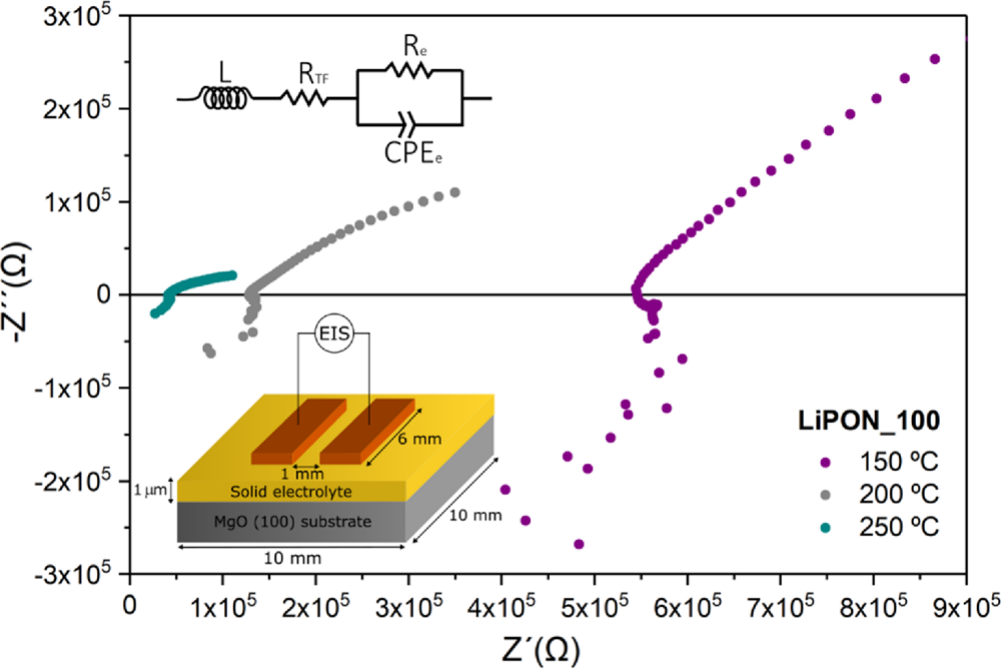
Obtained electrochemical impedance spectra of the LiPON_100 thin-film electrolyte at 150, 200, and 250 °C along with the used equivalent circuit and the schematic illustration of the setup for the electrical measurement of the film samples.

After characterizing the LiPON morphology and composition, the in-plane ionic conductivity of the thin films was measured by means of electrochemical impedance spectroscopy (EIS) using a two-probe method. Figure 5 shows the schematic illustration of the experimental setup for the electrical measurement of the thin-film conductivity, including the dimensions and layout of the Cu contacts. Exemplifying Nyquist plots obtained for a LiPON_100 film at different temperatures are also reproduced (the results obtained at 100 °C have not been graphed to show the other plots with better resolution). The corresponding equivalent circuit is shown in the figure inset, a setup-associated inductance (*L*) was always observed at high frequencies followed by a resistive contribution (*R*_TF_), in series with an *R*_e_–CPE_e_ contribution at low frequencies. The setup-associated inductance is displayed as negative Z″ values that correspond to a high-frequency inductive response of the measurement system (electrometer and wires) that cannot be eliminated.^50^ The series resistance *R*_TF_ is interpreted as the ohmic resistance associated with the Li^+^ conduction through the thin film, whereas the low-frequency contribution is associated with electrode-related phenomena. Moreover, note that the expected electronic resistance associated with contacts and cables, incorporated in the *R*_TF_ contribution, is much lower than that associated with the Li^+^ mobility and can thus be neglected.

The temperature dependence of the electrolyte resistivity is widely studied in the reported literature,^51,52^ being a thermally activated conduction mechanism that follows the Arrhenius equation (eq 3)^53^
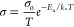
3where σ_o_ is the pre-exponential factor related to the number of charge carriers, *T* is the temperature in Kelvin, *k* is the Boltzmann constant, and *E*_a_ is the activation energy of the diffusion process.

**Figure 6:**
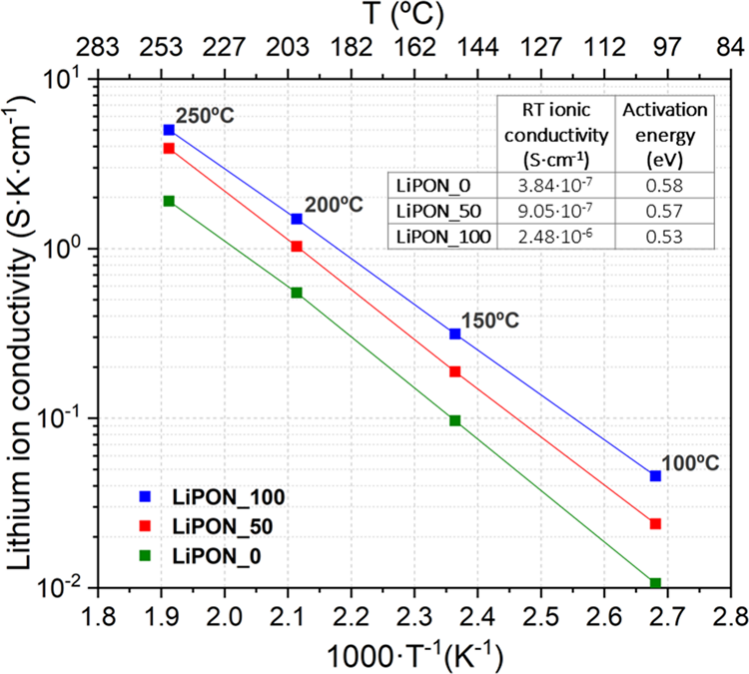
Arrhenius plots of ionic conductivity and activation energy at room temperature for samples LiPON_0, LiPON_50, and LiPON_100.

Figure 6 presents the Arrhenius representation of the ionic conductivity calculated from the measured impedance at different temperatures for the three samples under study. Two clear observations can be drawn from this figure. First, the ionic conductivity of LiPON significantly increases with the incorporation of nitrogen, reaching a maximum value of 9.56×10^–2^ S cm^–1^ at 250 °C for the LiPON_100 film. Second, the activation energy is slightly reduced, from 0.58 to 0.53 eV for the LiPON_0 and 100, respectively. These results match well with previous observations in the literature since N substitution in the Li_3_PO_4_ glass structure seems to reduce the non-bridging vs bridging P–O bond ratio, favoring the Li^+^ hopping through the LiPON network.^26,54^ Also, from a processing perspective, the results suggest that a N_2_-saturated background pressure during sputtering deposition is preferred for the incorporation of the optimum amount of N in the LiPON system. By extrapolating the ionic conductivity to room temperature (RT), the best-performing film, LiPON_100, reaches a conductivity of 2.48×10^–6^ S cm^–1^, in good agreement with the literature (see, *e.g.*, Bates and co-workers, who measured ∼2×10^–6^ S cm^–1^ at 25 °C in films deposited also by sputtering Li_3_PO_4_ in pure N_2_).^16^ These results validate, from a Li^+^-ion mobility perspective, the use of LiPON_100 as a solid electrolyte in this work.

### 3.2 Anode Characterization

**Figure 7:**
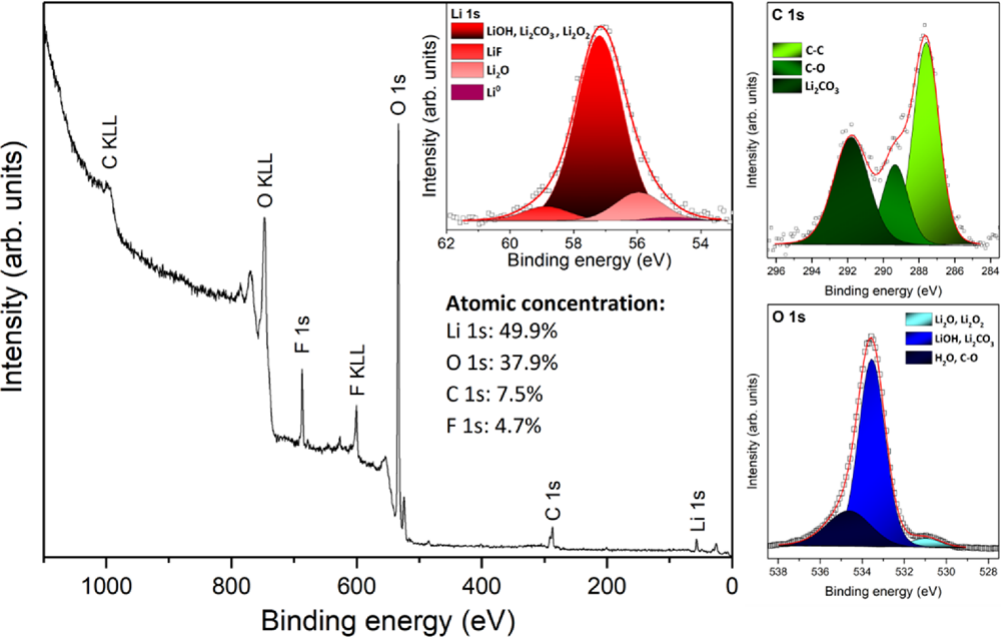
XPS survey spectra, Li 1s (inset graph), C 1s, and O 1s regions of the evaporated lithium deposited on the SS substrate (Mg Kα source). The normalized surface atomic concentration is also shown in the figure.

The lithium films were deposited on SS substrates and presented a granulated and highly dense structure, as can be seen in Figure S3 of the SI. Moreover, the lithium surface roughness is induced by the morphology of the SS substrate. The elemental characterization of the as-deposited lithium surface was carried out using XPS. Figure 7 shows the survey spectrum, along with the Li 1s, O 1s, and C 1s regions acquired with higher resolution. The atomic concentration values were calculated from the peak areas of each element in the survey spectrum, considering the corresponding relative sensitivity factors and the equipment transmission function. From obtained atomic concentration values, it can be concluded that the surface layer consists of lithium (49.9%), oxygen (37.9%) and, carbon (7.5%). Besides the adventitious carbon and surface oxides, some fluorine traces (4.7%) were also detected: most probably coming from the Viton gaskets used in the XPS chamber. According to the O 1s and C 1s regions, where Li_2_CO_3_ and Li_2_O/Li_2_O_2_ components appear, it can be concluded that the as-deposited anode surface mainly consists of oxides and carbonates. Based on these deconvolutions, it is also possible to identify the different species that are usually overlapping in the Li 1s region, namely, LiOH, Li_2_CO_3_, Li_2_O/Li_2_O_2_, LiF, and to a significantly lesser extent Li^0^. All these contaminants result from the reaction of Li with traces of O_2_, CO_2_, F, and H_2_O present in the process chamber even when not exposed to air.

The electrochemical performance of the LNMO//Li thin-film cell assembled with the conventional liquid electrolyte as a separator is added in the SI (Figure S4).

### 3.3 Electrochemical Performance of the All-Solid-State Cell

**Figure 8:**
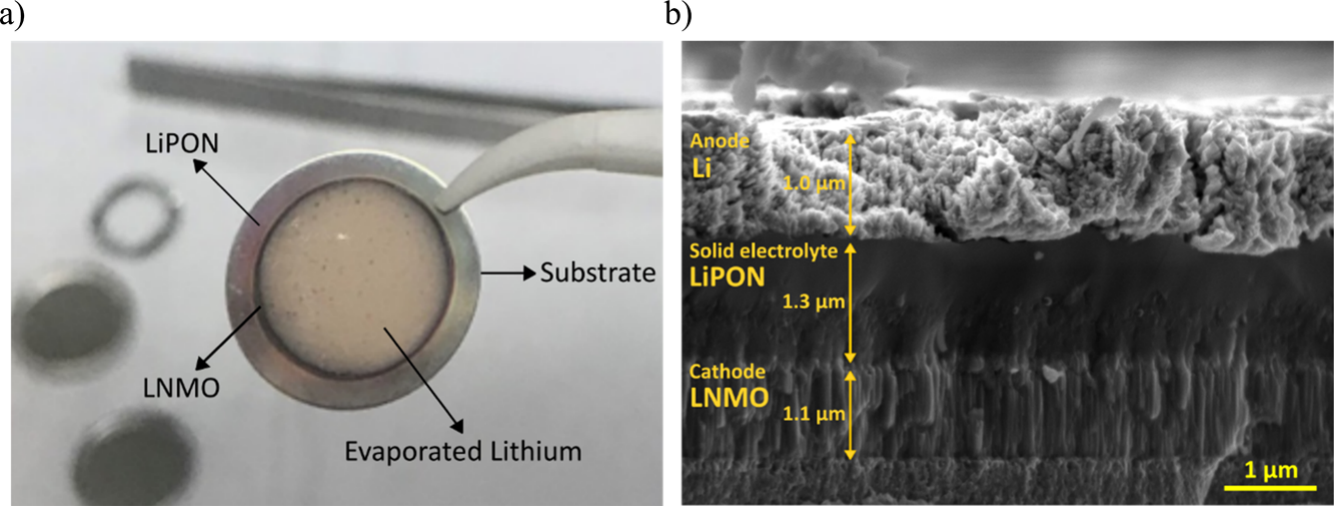
(a) Appearance of the ASSB full cell. Different layers can be identified depending on the diameter of the masks used to deposit them. (b) Cross-sectional SEM image of the battery stack deposited on a silicon substrate.

In Figure 8a, the ASSB stack is shown after deposition of all components, which can be clearly differentiated. Figure 8b shows the cross-sectional SEM image of the thin-film cell. The whole cathode/electrolyte/anode cell stack has a thickness below 5 μm. This particular cross section image was obtained using silicon as the substrate due to its handling advantages in terms of ease of cleavage (note that this substrate is not the one used for functional ASSBs). A columnar layer of LNMO with an approximate thickness of 1 μm and, on top of the latter, a smooth surface film of LiPON can be observed in Figure 8b. The good adhesion between the different defect-free and uniform thin films, with no evidence of any intermediate newly formed layer, can also be inferred from this figure. For the anode, a non-uniform surface was obtained. In addition, the Li thin film was covered with a titanium layer, deposited by DC magnetron sputtering, in order to avoid the interaction/degradation of Li metal under the SEM electron beam.

**Figure 9:**
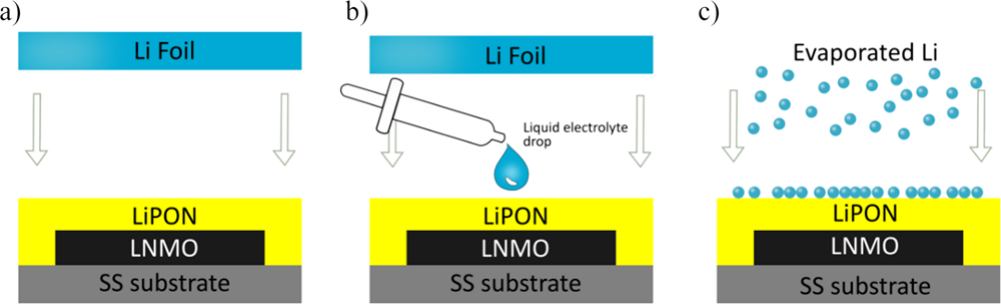
Schematic diagrams of the tested cell structures: (a) ASSB_Foil, (b) ASSB_Drop_Foil, and (c) ASSB_ ThinFilm.

Figure 9 shows a diagram of the three cells with different anode structures analyzed in this work: LNMO/LiPON/Li foil, LNMO/LiPON/Li foil + liquid electrolyte drop, and LNMO/LiPON/evaporated Li. They will be referred hereinafter as ASSB_Foil, ASSB_Drop_Foil, and ASSB_ThinFilm, respectively. It is important to note that considering the density and homogeneity of LiPON films (Figures 2 and 8 and Figure S1) along with the larger area of the LiPON film with respect to the LNMO one, the possibility of having direct contact of the liquid electrolyte with the LNMO is negligible.

**Figure 10:**
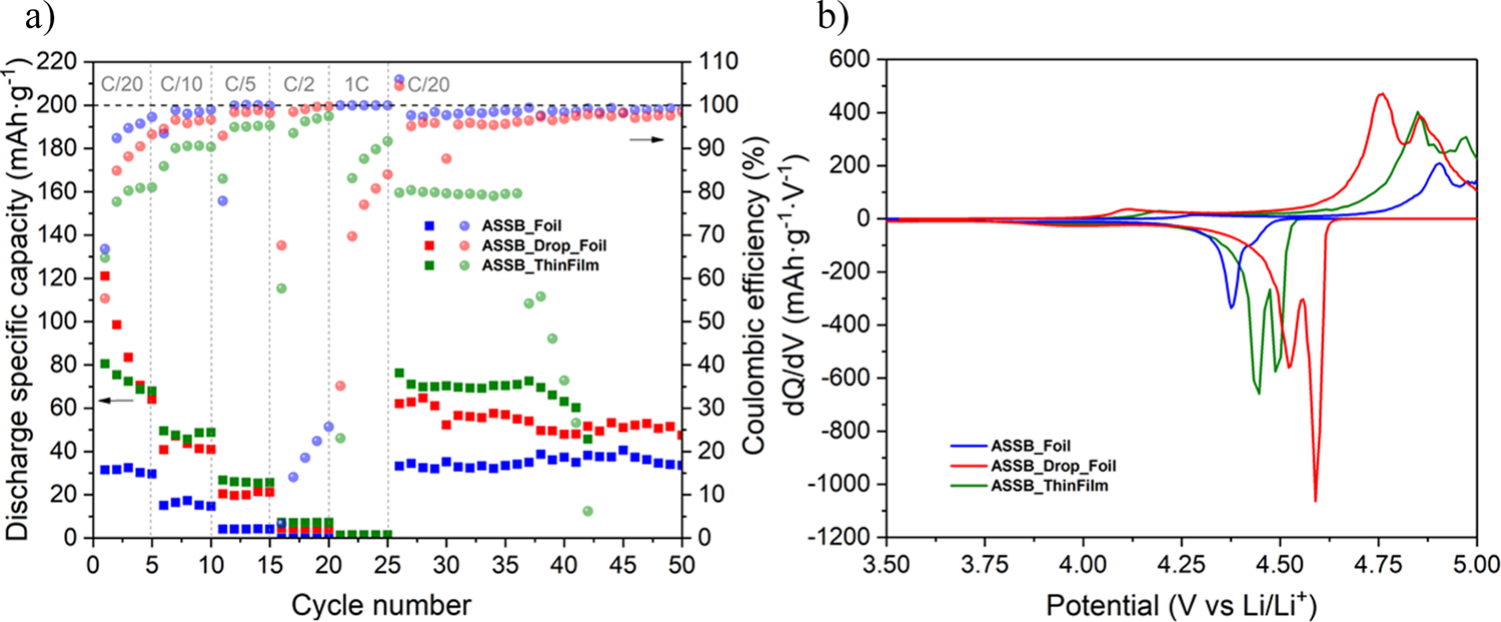
(a) Discharge specific capacity (filled squares) and corresponding Coulombic efficiency (filled circle) at different C-rates and (b) d*Q*/d*V* vs voltage curves derived from the charge/discharge cycle at C/20 of ASSB_Foil, ASSB_Drop_Foil, and ASSB_ThinFilm cells.

Figure 10a shows the discharge specific capacity and the corresponding CE of ASSB_Foil, ASSB_Drop_Foil, and ASSB_ ThinFilm at different C-rates (between 3.5 and 5 V vs Li/Li^+^). At C/20, the discharge specific capacity of ASSB_Foil was 33.0 mAh g^–1^, much lower than the other two samples. When the drop of liquid electrolyte is introduced, ASSB_Drop_Foil, the initial value at C/20 reached 121.1 mAh g^–1^. However, this value decreases rapidly over the first 5 cycles at this C-rate, losing 52.9% of its initial value. This may be due to the irreversible reactions triggered by the liquid electrolyte that occur at the interlayers during the first few cycles, leading to the formation of an SEI in the Li anode as suggested by the electrochemical response of the LNMO//Li cell assembled with the liquid electrolyte and shown in Figure S4. This deterioration was not that evident in the case of the thin-film cell, ASSB_ThinFilm, which in the fifth cycle only loses 15.6% of its initial value (80.5 mAh g^–1^). At C/10, the highest discharge specific capacity value corresponds to ASSB_ThinFilm, 48.8 mAh g^–1^, better than the 40.9 and 16.4 mAh g^–1^ of ASSB_Drop_Foil and ASSB_Foil, respectively. By increasing the C-rate to C/5, the ASSB_ThinFilm, ASSB_Drop_Foil, and ASSB_Foil cells retain 52.5, 51.8, and 25.0% of their capacities at C/10, respectively. The 85.2% is lost at C/2 in the thin-film case, the 89.3% in the lithium foil with drop case, and almost 100% in the lithium foil without drop case. Although in all three cells, the capacity is reduced down to zero at 1C, they recover 94.6, 52.9, and almost 100% (ASSB_ThinFilm, ASSB_Drop_Foil, and ASSB_Foil, respectively) of their initial specific discharge capacities upon cycling back to C/20 (note that these values were calculated based on the first cycle at C/20). In the last long cycling at C/20, the discharge specific capacity values (ASSB_ThinFilm > ASSB_Drop_Foil > ASSB_Foil) remain relatively stable. As is can be seen, the liquid electrolyte drop enhances ion diffusion between the lithium foil and the LiPON. Even so, the result can be improved by using evaporated lithium. Unfortunately, in cycle 37, the ASSB_ThinFilm begins to lose the specific discharge capacity, short-circuiting the battery. The formation of lithium dendrites may be the cause of this deterioration.

Regarding the CE, rather low values were obtained in the first cycle, particularly in the case of the ASSB_Drop_Foil (55.4%, versus 64.8 and 66.8% of ASSB_ThinFilm and ASSB_Foil, respectively). This is ascribed to the liquid electrolyte decomposition process in the anode interface at high voltages occurring predominantly during the charge.^55^ The first cycle has been included in the SI. The LiPON, besides its role as a solid electrolyte, also acts as a cathode protector.^31,32^ For the rest of the cycle, ASSB_Foil shows a CE of 97.3% in the last cycle at C/20, exceeding 98% at C/10 and reaching almost 100% at C/5. ASSB_Drop_Foil was below 94% in the last cycle at C/20, while the CE was 96.6 and 98.8% at C/10 and C/5, respectively. Furthermore, higher CE values for the ASSB_Foil than for the ASSB_Drop_Foil were observed during long cycling at C/20: 98.9 and 97.3%, respectively. In the case of ASSB_ThinFilm, the CE remains below the other two cells, up to 81.0% during the initial cycling stage at C/20, reaching a maximum value of 97.4% at high C-rates (C/2) and around 80% during the last long cycling at C/20. A possible explanation for the pronounced difference observed in the CE may likely rely on the high reactivity of the evaporated lithium with the solid electrolyte; in addition, more unwanted reactions are expected for ASSB_ThinFilm cells than for ASSB_Foil cells due to the high surface-to-volume ratio, typical of thin-film electrodes.

The ASSB_ThinFilm cell electrochemical performance is limited, particularly if it is compared with a previous report of the same cell grown by RF sputtering on Pt-coated Al_2_O_3_ substrates.^6^ In this case, the cell delivered a first cycle capacity of 122 mAh g^–1^ at C/10 and a 90.6% capacity retention after 10,000 cycles and a 99.98% Coulombic efficiency. During the revision process of this work, another report on thin-film LNMO/LiPON/Li cells grown by pulsed laser deposition (PLD) on Pt-coated Al_2_O_3_ substrates was published.^56^ This work presented a cell that delivered 99.6% Coulombic efficiency after 600 cycles; however, the reported capacity values of the cell were not very high: ∼18 μAh cm^–2^ μm^–1^ as normalized to the cathode thickness, three times lower than the capacity of our cathode (65 μAh cm^–2^ μm^–1^).^57^ In any case, it is worth mentioning that those two reports on thin-film LNMO/LiPON/Li cells present results of batteries that have been grown on Pt-coated Al_2_O_3_ substrates, with the Pt cost at $28,000 kg^–1^, using evaporation techniques, namely, RF magnetron sputtering and PLD, which involve a much higher production cost than the ones used in this work.

In order to analyze the redox reactions and voltage hysteresis between the charge and discharge of the battery, differential capacity curves (d*Q*/d*V*) were used (Figure 10b). In all three cells, during charge (from 3.5 to 5 V vs Li/Li^+^) and discharge (from 5 to 3.5 V vs Li/Li^+^), two high-voltage transitions can be distinguished related to the successive oxidation of Ni^2+^ to Ni^3+^ and Ni^4+^. In addition, another capacity contribution corresponding to the presence of Mn^3+^ (redox pair Mn^3+^/Mn^4+^) at around 4 V vs Li/Li^+^ is also revealed, particularly in the ASSB_Drop_Foil cell. In the case of ASSB_Foil, the nickel-related redox peaks appear during the charge at 4.90 and 4.97 V vs Li/Li^+^ (Ni^2+/3+^ and Ni^3+/4+^, respectively), while, during discharge, they appear at 4.37 and 4.43 V vs Li/Li^+^, the second transition being less pronounced than the first one. This voltage difference leads to a hysteresis of 0.53 V, higher than in the other two cells: 0.26 V for the ASSB_Drop_Foil cell and 0.39 V for the ASSB_ThinFilm cell. These results prove that the liquid electrolyte drop at the interface between the Li foil anode and LiPON electrolyte can effectively weaken the polarization during charge and discharge, most probably due to an improvement of the lithium-ion migration. Importantly, the evaporated lithium reduces this polarization without the need of introducing any liquid component, thus improving battery safety.

**Figure 11:**
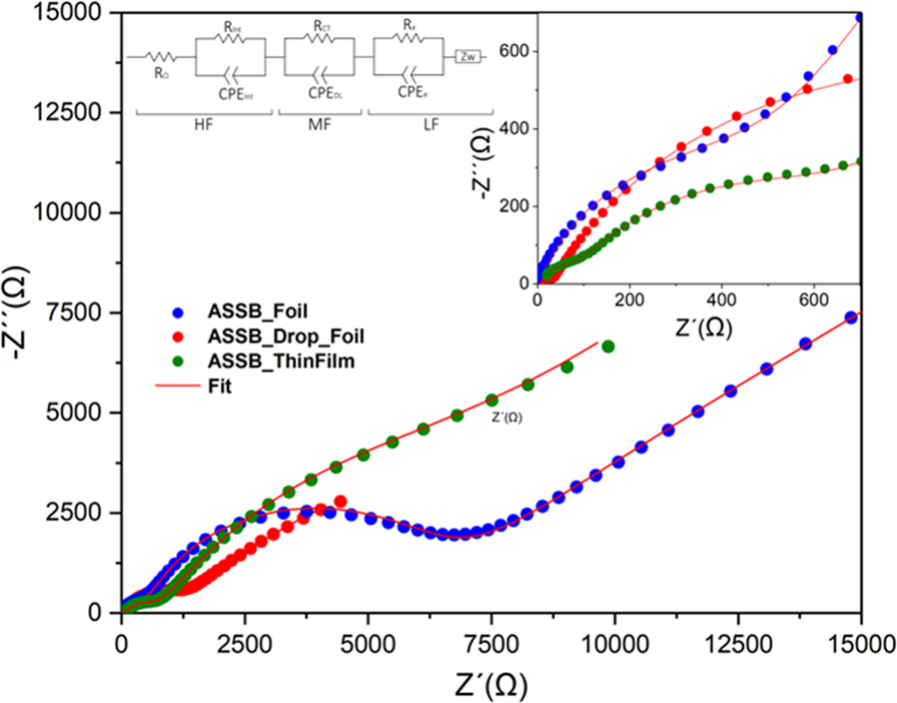
Nyquist plots of ASSB_Foil, ASSB_Drop_Foil, and ASSB_ThinFilm along with the equivalent circuit and the fitted data.

For a better analysis of the internal resistance, an EIS study was carried out. The data were collected in the 100 mHz–7 MHz frequency range once the cell was assembled. Figure 11 shows the Nyquist plots of ASSB_Foil, ASSB_Drop_Foil, and ASSB_ThinFilm cells fitted with the proposed equivalent circuit (see inset drawing). For the sake of clarity, the zoomed graph is included in the right-inset graph. The impedance curves show a semicircle at a high frequency (HF) followed by a more prominent semicircle at mid frequency (MF) and ending with a semicircular tail at low frequency (LF). A possible explanation of the equivalent circuit could be as follows: the HF contribution is originated by the *R*_int_–CPE_int_ contribution of interfacial layers in series with the electrolyte ohmic resistance (*R*_Ω_), corresponding to the Li^+^ resistance across the electrolyte (the expected electronic resistance associated with contacts and cables was neglected). The semicircle at MF is ascribed to a charge-transfer resistance and a double-layer capacitance (*R*_CT_–CPE_DL_), both connected in parallel. Finally, the LF range is ascribed to an RC semicircle associated with bulk electronic resistance (*R*_e_) and charge accumulation capacitance (CPE_e_) along with the Warburg diffusion element (Zw) that accounts for the sloping line ascribed to the solid-state diffusion of Li^+^ in the crystal. Table 2 presents the fitted values for the high-frequency part, which is the one ascribed to the interface processes. Since different samples are involved, the results have been normalized concerning the area for resistance (area-specific resistance, ASR) in Ω cm^2^ and capacity in F cm.

**Table 2:** Values Corresponding to the Interfacial Layer (*R*_int_–*C*_int_) Contribution, Charge-Transfer Resistance and a Double-Layer Capacitance (*R*_CT_–*C*_DL_), and Respective Time Constants (τ = *RC*)

	ASSB_Foil	ASSB_Drop_Foil	ASSB_ThinFilm
*R*_int_ (Ω cm^2^)	530	30	105
*C*_int_ (F cm^–2^)	3.2 × 10^–9^	2.6 × 10^–8^	1.1 × 10^–8^
τ_int_ (s)	1.7 × 10^–6^	7.8 × 10^–7^	1.2 × 10^–6^
*R*_CT_ (Ω cm^2^)	6,198	1,070	697
*C*_DL_ (F cm^–2^)	2.8 × 10^–8^	4.3 × 10^–6^	1.8 × 10^–7^
τ_CT–DL_ (s)	1.7 × 10^–4^	4.6 × 10^–3^	1.3 × 10^–4^

The different RC contributions of the three cells could be compared considering the value of τ. In the first contribution, the three samples are roughly of the same order of magnitude (10^–6^), suggesting that they correspond to the same interfacial feature. Particularly, they refer to the formation of a passivating layer on the electrode/electrolyte interface. The value of the resistance (*R*_int_) follows this trend for the different cells: ASSB_Drop_Foil < ASSB_ThinFilm ≪ ASSB_Foil. In the ASSB_Foil cell, the poor contact between the lithium and the solid electrolyte makes the formation of the SEI layer between them highly resistive. By improving the contact by adding a liquid electrolyte, the *R*_int_ was reduced to 30 Ω cm^2^, resulting in a significant improvement of the SEI. The ASSB_ThinFilm cell achieved an intermediate resistance, exactly 105 Ω cm^2^. The interfacial reactions between the evaporated lithium anode and the solid electrolyte seem to be more active than those corresponding to the lithium foil. This high reactivity, and the absence of contaminating species that are typically present on the surface of commercial lithium foil, helps the formation of a better SEI, thus improving contact.

The τ of the second semicircle also coincides in all three samples (∼10^–4^ s). Interestingly, a lower charge-transfer resistance was obtained in the case of the evaporated lithium, 698 Ω cm^2^ vs 1,070 and 6,199 Ω cm^2^ for ASSB_Drop_Foil and ASSB_Foil cells, respectively. This improvement in the pass through of Li ions could be caused by a larger contact surface. The fact that lithium is deposited directly on the LiPON surface, without the need to introduce a bonding medium, results in a more active area. The higher resistance of the ASSB_Drop cell may also be due to the fact that there are two media through which the Li must pass (two charge transfers; from Li ions to liquid and from liquid to LiPON). In the case of ASSB_Foil, the roughness and stiffness of the Li foil make the active surface smaller, which would be behind the highest charge-transfer resistance observed.

**Figure 12:**
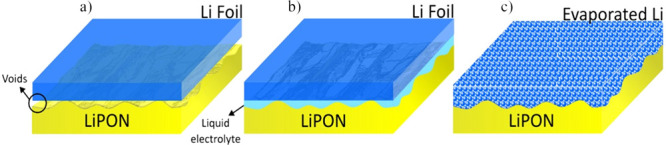
Schematic illustration of the anode/solid electrolyte interface of (a) ASSB_Foil, (b) ASSB_Drop_Foil, and (C) ASSB_ThinFilm.

For a better interpretation, Figure 12 shows a schematic representation of the anode/electrolyte interface of ASSB_Foil, ASSB_Drop_Foil, and ASSB_ThinFilm cells. In the ASSB_Foil case, where the lithium foil is placed directly on top of the solid electrolyte, the Li-ion diffusion paths are limited by the voids created in the interface due to the poor adhesion between the Li foil and the LiPON.^37^ When a drop of liquid electrolyte is added, the voids are filled, improving their contact although adding an extra media for the Li to cross. For the ASSB_ThinFilm cell, the lithium was evaporated, avoiding possible voids due to the adaptation of the lithium atoms to the roughness of the LiPON.

Finally, to validate the successful implementation of the monolithic high-voltage all-solid-state thin-film battery deposited layer by layer, a series of voltage tests were performed. Figure S7b of the Supporting Information shows the nominal voltage of the assembled high-voltage pouch cell delivering 4.72 V. Moreover, the cell was able to light two 2.1 V forward voltage red LEDs arranged in series (Figure S7c of the Supporting Information). This is something that cannot be done with a conventional Li-ion battery as shown in the comparative experiment shown in the movie uploaded as a web-enhanced object where the lighting of the two 2.1 V red LEDs was attempted by a commercial Li-ion battery and our high-voltage battery (Video S1).

## 4 Conclusions

In this work, a functional high-voltage, all-solid-state thin-film lithium-ion battery composed of LNMO as the cathode, LiPON as the solid electrolyte, and an evaporated lithium anode has been deposited layer by layer on a low-cost stainless-steel current collector. At $1.5 kg^–1^, the cost of stainless steel is five times lower than Cu at $8 kg^–1^ and three orders of magnitude lower than Pt at ∼$28,000 kg^–1^. Considering that stainless steel is already commercially available in >20 μm-thick foils, there can be a great saving with respect to conventional cells using Cu as the current collector or other thin-film cells that rely on 0.65 μm-thick Pt layers, which have to be supported on an additional substrate. In terms of processing techniques, the AC magnetron sputtering technique used for the cathode growth is an effective physical vapor deposition technique that has several advantages over its RF magnetron sputtering counterpart. Besides improving process stability and increasing deposition rates, it is a processing technique that is used at lower power levels, with a low degree of technical complexity, hence resulting in a cost-effective processing method. The main cost difference is found on the power supply, which can range from $4,000 for a 10 kW AC power supply to $3,000 for a 600 W RF power supply, again several orders of magnitude difference in favor of AC magnetron sputtering, which needs lower maintenance and can reach deposition rates on oxide materials up to 100 nm min^–1^, while RF magnetron sputtering yields deposition rates of 1 nm min^–1^. Altogether, it represents a considerable cost reduction when using AC magnetron sputtering.

Before assembling the cells, each electroactive component of the battery has been characterized separately. All the deposited LiPON samples show smooth and highly dense structures with no signs of crystallization. The high impedance at RT evidenced the need for higher temperatures for a proper analysis of the LiPON ionic conductivity by means of the Arrhenius equation. The ionic conductivity increased with the temperature, being higher in samples deposited with a larger nitrogen percentage. As a result, the higher ionic conductivity at RT of 2.48×10^–6^ S cm^–1^ corresponds to the N_2_-saturated sputtering process, in good accordance with the literature. Moreover, the XPS shows a broad signal that can be resolved with N_d_ and N_t_ components and the analysis matches well with the proposed O-substitution theoretical models, suggesting efficient nitrogen incorporation. All in all, this indicates that a N_2_-saturated background pressure is preferred for the incorporation of the optimum amount of N in the LiPON solid electrolyte system. Lithium anode thin films of approx. 1 μm were prepared by thermal evaporation on a stainless-steel substrate. The deposited lithium thin films presented a granulated and highly dense structure, with a substrate-induced roughness. Because of the high reactivity of the evaporated lithium, the surface mainly consists of oxides and carbonate species. Finally, the build-up of a high-voltage all-solid-state thin-film battery by combining the optimized functional films (electrode and electrolyte) was performed, sequentially depositing on a stainless-steel substrate.

To validate the successful implementation of the thin-film battery, a comparison of the electrochemical performance of the microbattery using evaporated lithium as well as lithium foil (commonly employed) was done. The lack of contact between the lithium foil and the LiPON limits the diffusion of ions between the anode and the electrolyte, thereby impairing the electrochemical performance of the cell. The addition of a liquid electrolyte drop provides a homogeneous interface, reaching 121.12 mAh g^–1^ at C/20. The reduced cycle life of the evaporated lithium cell could have its origin in the Li film that presents a rougher morphology than the LNMO and LiPON layers. The low Coulombic efficiency, between 80 and 95%, of the evaporated lithium cell, in contrast with the higher values in the 95–99.9% range observed for the Li foil cell, is ascribed to interface reactions between LiPON and evaporated Li, which is highly reactive on its atomic form while being helped by the typically enhanced high surface-to-volume ratio of thin films. In any case, the evaporated lithium cells reach a higher specific capacity after 25 cycles, >70 mAh g^–1^, for the evaporated Li cell, in contrast with the values below 60 mAh g^–1^ when using Li foil plus liquid electrolyte and the values below 40 mAh g^–1^ when using Li foil. These results indicate a significant progress in the good direction toward the consecution of a commercial high-voltage thin-film battery supported on low-cost and abundant stainless-steel substrates.

## Supporting Information Available

(Section S1) Top-view SEM images of LiPON thin films deposited with different Ar-N_2_ content, (Section S2) partial structure of nitride Li_3_PO_4_ thin film, (Section S3) SEM images of the evaporated lithium on the SS substrate sample, (Section S4) C-rate of the LNMO//Li thin film cell assembled with the conventional liquid electrolyte as a separator, (Section S5) EDS mapping of the ASSB, (Section S6) initial cycle and differential capacity curves of the ASSB cells, and (Section S7) ASSB encapsulation system and validation (PDF)
Lighting of red LEDs using a commercial Li-ion battery and the high-voltage battery in the study (AVI)

## Supplementary Material

ae2c01581_si_001.pdf

ae2c01581_si_002.avi
