# Supplementary material for: Monolithic All-Solid-State High-Voltage Li-Metal Thin-Film Rechargeable Battery
Source: ACS Appl Energy Mater. 2022 Sep 27;5(10):12120–31. doi: 10.1021/acsaem.2c01581 (PMC9603062; doi:10.1021/acsaem.2c01581)
Supplement: Supplementary file 1 — ae2c01581_si_001.pdf [file ae2c01581_si_001.pdf]

Supporting Information

Monolithic all-solid-state high voltage Li-metal thin-film rechargeable battery

Iñaki Madinabeitia[†,‡,§], Jokin Rikarte[‡,§], Ane Etxebarria[‡,§,∇], Giorgio Baraldi[2,||], Francisco José Fernández-Carretero[†], Iñigo Garbayo[‡,⊥], Rosalía Cid[‡], Alberto García-Luis[†], and Miguel Ángel Muñoz-Márquez[‡,#,]*

† TECNALIA, Basque Research and Technology Alliance (BRTA), Parque Científico y Tecnológico de Gipuzkoa, Mikeletegi Pasealekua 2, 20009 Donostia-San Sebastián, Spain

‡ Centre for Cooperative Research on Alternative Energies (CIC energiGUNE), Basque Research and Technology Alliance (BRTA), Alava Technology Park, Albert Einstein 48, 01510 Vitoria-Gasteiz, Spain

§ Departamento de Física de la Materia Condensada, Facultad de Ciencia y Tecnología, Universidad del País Vasco, UPV/EHU, P.O. Box 644, 48080 Bilbao, Spain

Corresponding Author

Miguel Ángel Muñoz-Márquez: miguel.munoz@unicam.it

Present Addresses

School of Science and Technology – Chemistry Division, University of Camerino. Via Madonna delle Carceri - 62032 Camerino, Italy

∇ Interface Science Department. Fritz Haber Institute of the Max Planck Society, Faradayweg 4-6, 14195 Berlin, Germany

|| CIDETEC, Basque Research and Technology Alliance (BRTA), Parque Científico y Tecnológico de Gipuzkoa, Miramon Pasealekua 196, 20014 Donostia-San Sebastián, Spain

⊥ CENER (National Renewable Energy Centre), Av. Ciudad de la Innovación 7, 31621 Sarriguren, Spain

S1. Top-view SEM images of LiPON thin films deposited with different Ar-N_2 content

Figure S1 shows the top-view SEM images of the LiPON_0, LiPON_50, and LiPON_100 films. Some small differences could be observed in the morphology. While the surface of the sample deposited only with argon, Figure S1.a, is granulated, a smoother surface was obtained with increasing nitrogen content (Figure S1.b,c). Nevertheless, from a morphological point of view, all films presented the desired dense and homogeneous structure required.

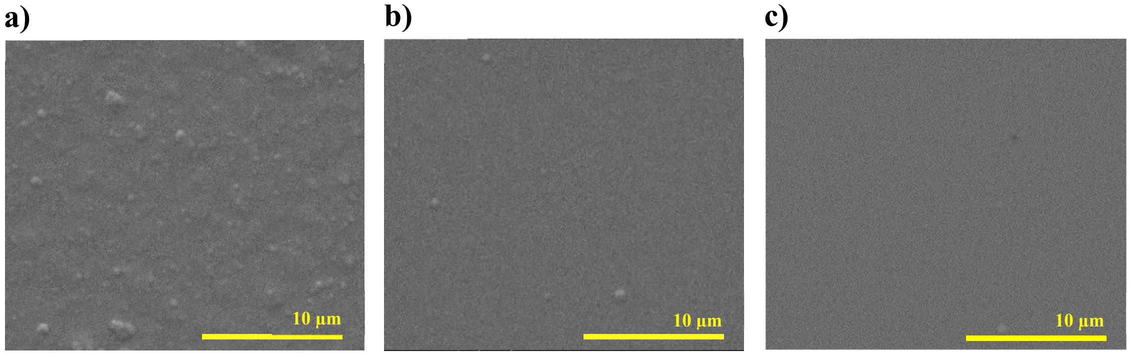

Figure S1: Top-view SEM images of LiPON thin films deposited with different Argon-nitrogen content a) LiPON_0, b) LiPON_50, and c) LiPON_100, respectively.

S2. Partial structure of nitride Li_3PO_4 thin-film

Figure S2 shows the evolution of the partial structure of Li_3PO_4 glass after nitridation, incorporating to the structure >N- and -N=.

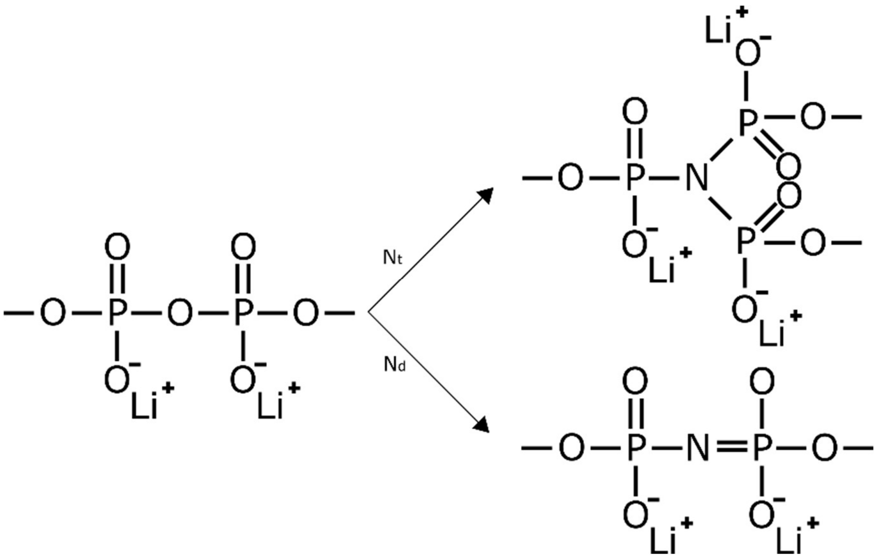

Figure S2: Partial structure of nitride Li_3PO_4 thin-film glass with the incorporation of –N< and –N=.

S3. SEM images of the evaporated lithium on SS substrate sample.

The morphology of the deposited lithium metal films was evaluated by means of top view SEM, as shown in Figure S3.a. The lithium films were deposited on SS substrates and presented a granulated and highly dense structure. The lithium surface roughness is induced by the morphology of the SS substrate, Figure S3.b.

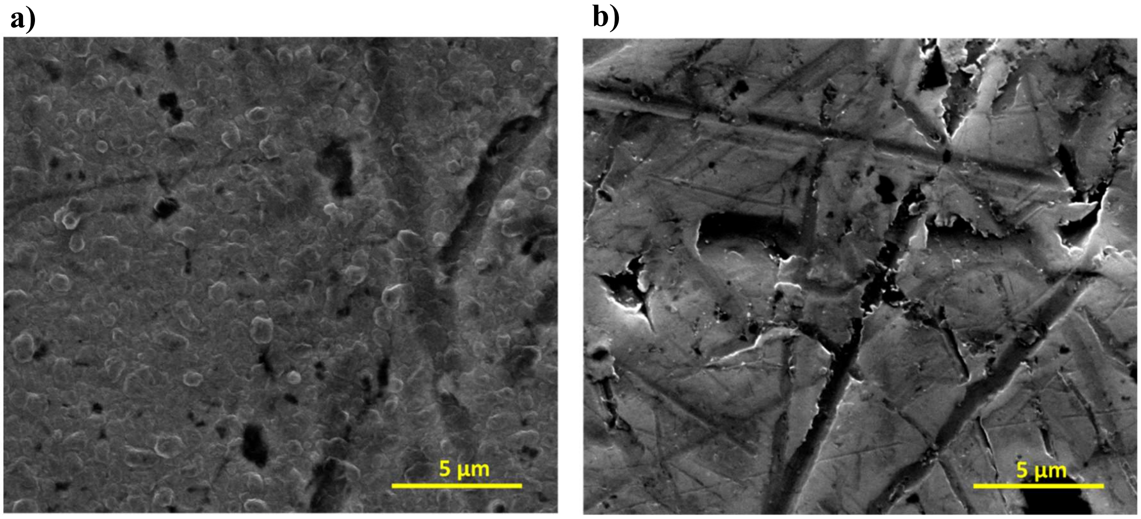

Figure S3: Top view SEM images of a) the evaporated lithium on SS substrate sample and b) the SS substrate.

S4. C-rate of the LNMO//Li thin film cell assembled with the conventional liquid electrolyte as separator.

For the electrochemical analysis, the lithium was evaporated on SS substrate (using 14 mm Ø mask) and was mounted in an air-tight CR2032 coin cell against the already characterized LNMO thin film and with a 1M of $LiPF_6$ in EC:DMC (1:1, v/v) liquid electrolyte in a glass fiber as separator. Figure S2 shows the rate capability and the corresponding CE of the evaporated lithium in the range from C/20 to C, and back to C/20. At C/20 the discharge specific capacity was 113.9 $mAh \cdot g^{-1}$, indicating that, at low C-rates, the Li-ion insertion through the LNMO does not depend on the used anode. At C/10 and C/5, the capacity retention observed was 91.6% and 85.0%, respectively. However, as the C-rate increases to C/2, the discharge specific capacity drops abruptly from 96.9 $mAh \cdot g^{-1}$ to 27.1 $mAh \cdot g^{-1}$. At 1C, the specific discharge capacity has lost 89.0% of its initial value. Moreover, the initial capacity is not recovered when cycled back to C/20, where only 23.9 mAh·g-1 are delivered. This value decreases as it cycles at C/20, reaching a value of 9.7 $mAh \cdot g^{-1}$ in the 15th cycle at C/20 (cycle 40 in total). The unstable anode-electrolyte interface may be behind the underperformance of the evaporated lithium. The same reactions affecting the lithium foil are also expected for evaporated lithium, but in this case, these reactions can affect the whole thin film. This reduction of active lithium is also confirmed by the CE evolution, which follows a similar trend as with lithium foil, starting from low efficiency in the first cycles (39.5%) and reaching values >97% at C/5. At C/2, along with the capacity

decay, the CE becomes unstable, remaining at values of 89.9% and 85.5% at the final C/20. Additionally, the non-uniformity of the anode, could contribute to this fast degradation. Notwithstanding, the integration of the solid electrolyte is expected to reduce the unwanted reactions between the organic liquid electrolyte and the lithium anode, thus improving its electrochemical

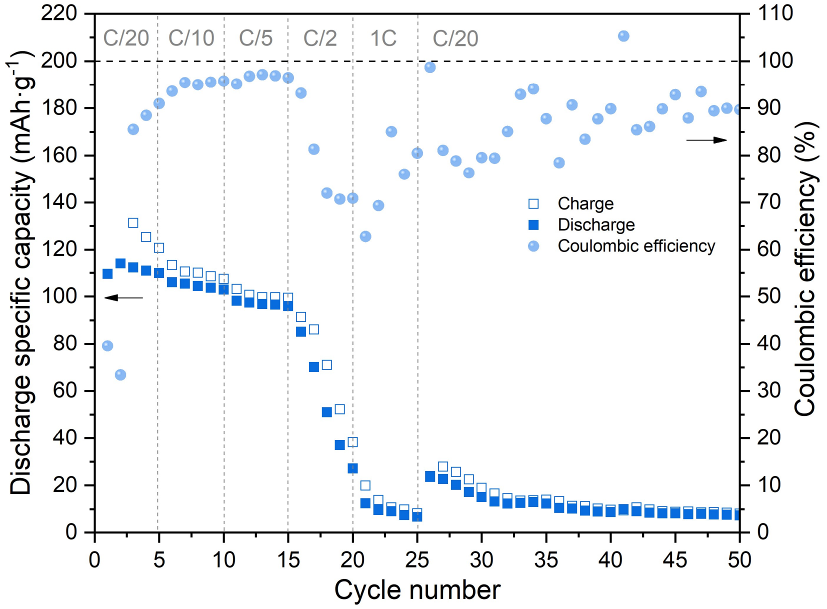

performance.

Figure S4: Charge (hollow squares), discharge (filled squares) specific capacities, and corresponding coulombic efficiency (filled circle) at different C-rates for LNMO//Li thin film cells assembled with conventional liquid electrolyte and separator.

S5. EDS mapping of the ASSB

EDS mapping results are presented in Figure S5. The detected elements were Si, Cr, N, Ni, Mn, P, and O (Li cannot be detected with this technique). Note that in this case a CrN interlayer has been used between the cathode and the silicon substrate that will act as a current collector. Silicon signal appears in the bottom part of the image and corresponds to the substrate used for the elemental/cross-sectional analysis. The first deposited layer is CrN, where it is easily differentiated by the Cr-rich layer on the top of the Si-wafer. In the intermediate layer, Ni and Mn signals are detected, along with more oxygen, related to the uniform LNMO layer. Some diffusion of the elements towards the anode can be observed. When lithium is deposited on the surface of the solid electrolyte, it tends to react with each of the elements present (P, O, and N), leading to the formation of Li_3PO_4, Li_3P, Li_3N, and Li_2O.

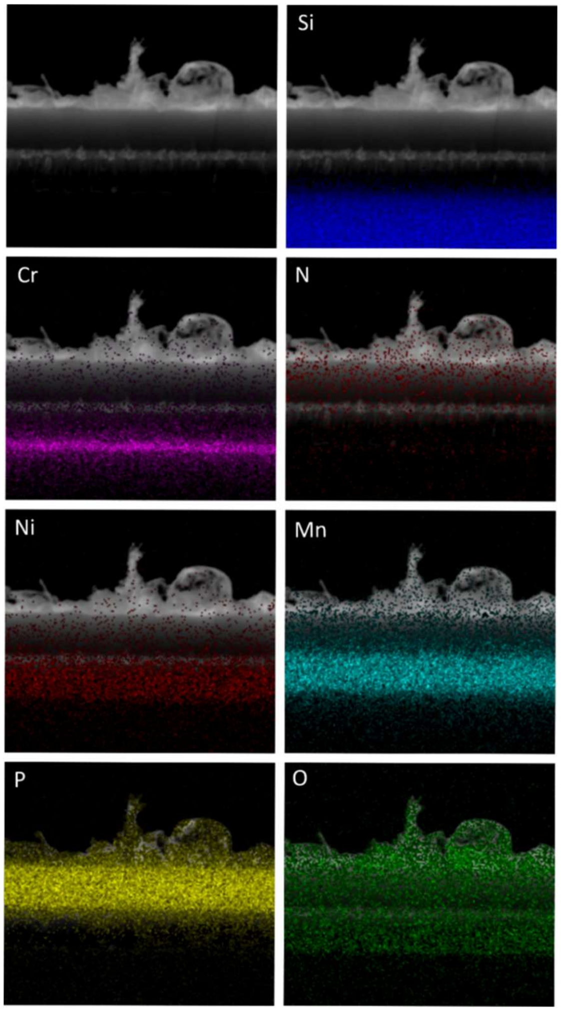

Figure S5: EDS mapping images obtained for Si, Cr, N, Ni, Mn, P, and O in the ASSB cross-section analysis.

S6. Initial cycle and differential capacity curves of the ASSB cells

Figure S6 shows the initial charge and discharge curves (between 3.5 to 5 V vs Li/Li^{+}) of the ASSB_Foil, ASSB_Drop_Foil and ASSB_ThinFilm cells. Two high voltage plateaus can be distinguished related to $Ni^{2+/3+}$ and $Ni^{3+/4+}$. Being clearer in the case of ASSB_Drop_Foil and ASSB_ThinFilm. Moreover, another plateau can be observed corresponding to the presence of Mn^{3+} (redox pair $Mn^{3+/4+}$) at around 4 V vs Li/Li^{+}, particularly in ASSB_Drop_Foil cell.

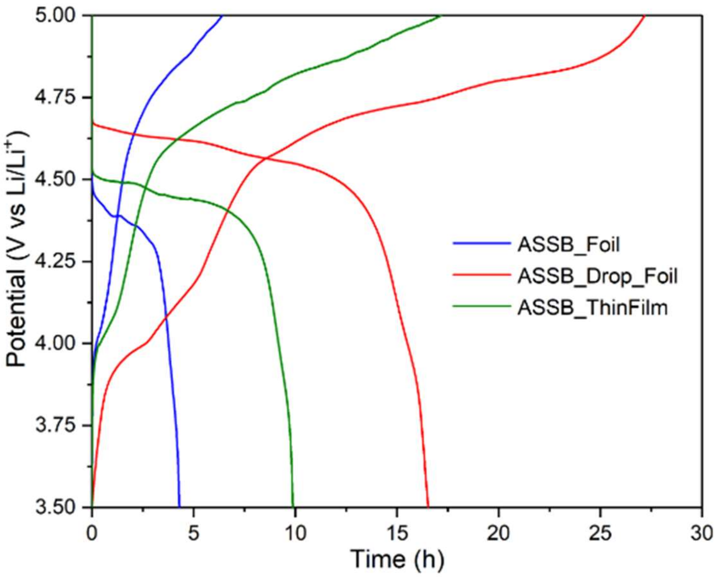

Figure S6: The initial cycle measured at C/20 of ASSB_Foil, ASSB_Drop_Foil, and ASSB_ThinFilm cells.

S7. ASSB encapsulation system and validation

In order to improve the electrical contacts, the ASSB cell was assembled in a pouch cell system, see Figure S7a. The used electrical contacts were aluminum for the cathode and nickel for the anode. Figure S7b shows the nominal voltage of this high voltage cell. Moreover, it was confirmed that the battery was able to light two 2.1 V vs Li/Li+ forward voltage red LEDs arranged in series, see Figure S7c.

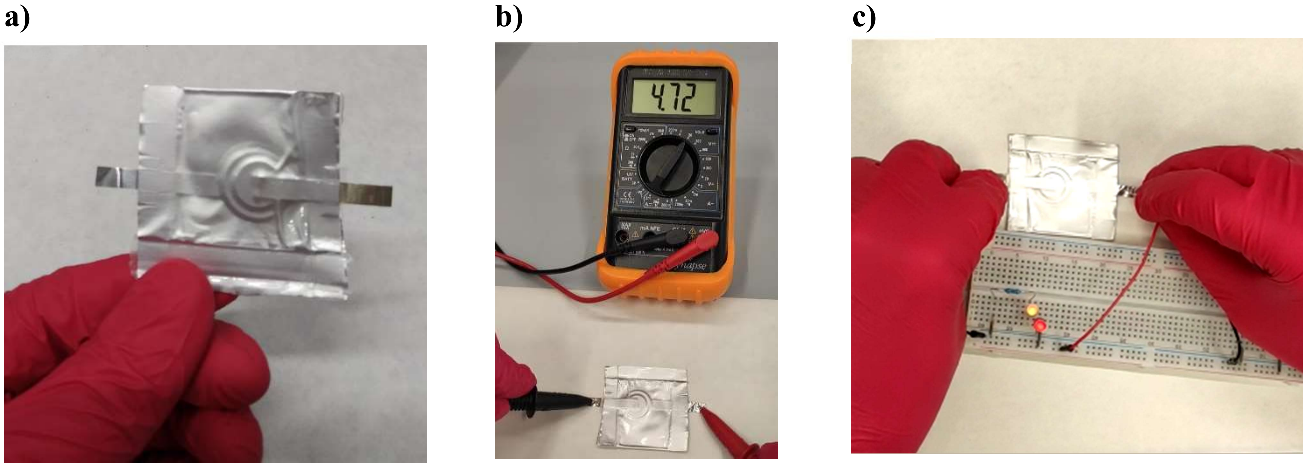

Figure S7: a) ASSB pouch cell system, b) nominal voltage, c) validation of its high nominal voltage.
